# Transcriptome Analysis Provides Insights into Catalpol Biosynthesis in the Medicinal Plant *Rehmannia glutinosa* and the Functional Characterization of *RgGES* Genes

**DOI:** 10.3390/genes15020155

**Published:** 2024-01-24

**Authors:** Yuanjun Li, Xiaoru Zhai, Ligang Ma, Le Zhao, Na An, Weisheng Feng, Longyu Huang, Xiaoke Zheng

**Affiliations:** 1 College of Pharmacy, Henan University of Chinese Medicine, Zhengzhou 450046, China; liyuanjun@hactcm.edu.cn (Y.L.);; 2 National Nanfan Research Institute (Sanya), Chinese Academy of Agricultural Sciences, Sanya 572024, China; 3 State Key Laboratory of Cotton Biology, Institute of Cotton Research of Chinese Academy of Agricultural Sciences, Anyang 455000, China; 4 Hainan Yazhou Bay Seed Laboratory, Sanya 572024, China

**Keywords:** transcriptome, *Rehmannia glutinosa*, catalpol, biosynthesis, geraniol synthase

## Abstract

*Rehmannia glutinosa*, a member of the Scrophulariaceae family, has been widely used in traditional Chinese medicine since ancient times. The main bioactive component of *R. glutinosa* is catalpol. However, the biogenesis of catalpol, especially its downstream pathway, remains unclear. To identify candidate genes involved in the biosynthesis of catalpol, transcriptomes were constructed from *R. glutinosa* using the young leaves of three cultivars, Beijing No. 3, Huaifeng, and Jin No. 9, as well as the tuberous roots and adventitious roots of the Jin No. 9 cultivar. As a result, 71,142 unigenes with functional annotations were generated. A comparative analysis of the *R. glutinosa* transcriptomes identified over 200 unigenes of 13 enzymes potentially involved in the downstream steps of catalpol formation, including 9 genes encoding UGTs, 13 for aldehyde dehydrogenases, 70 for oxidoreductases, 44 for CYP450s, 22 for dehydratases, 30 for decarboxylases, 19 for hydroxylases, and 10 for epoxidases. Moreover, two novel genes encoding geraniol synthase (RgGES), which is the first committed enzyme in catalpol production, were cloned from *R. glutinosa*. The purified recombinant proteins of RgGESs effectively converted GPP to geraniol. This study is the first to discover putative genes coding the tailoring enzymes mentioned above in catalpol biosynthesis, and functionally characterize the enzyme-coding gene in this pathway in *R. glutinosa*. The results enrich genetic resources for engineering the biosynthetic pathway of catalpol and iridoids.

## 1. Introduction

*R. glutinosa* (Dihuang), a medicinal herb from the Scrophulariaceae family, has extensive usage in traditional Chinese medicine for nourishing *Yin* and tonifying the *kidney*, and it is considered a “top-grade” herb [1]. Pharmacological research has revealed that *R*. *glutinosa* exerts positive effects on the blood system, endocrine system, nervous system, cardiovascular system, and immune system, such as hemostasis, anti-tumor, and immuno-enhancing activities [1]. Many kinds of compounds have been isolated from *R*. *glutinosa*, including iridoids, phenylethanoid glycosides, amino acids, and flavonoids, with iridoids being the most abundant metabolites. Catalpol, the main active iridoid glycoside of *R. glutinosa*, has been reported to possess various medicinal activities, such as neuroprotective [2], anti-inflammatory [3], anti-diabetes [4], anti-depressive [5], anti-oxidative [6], and anti-tumor effects [7].

**Figure 1:**
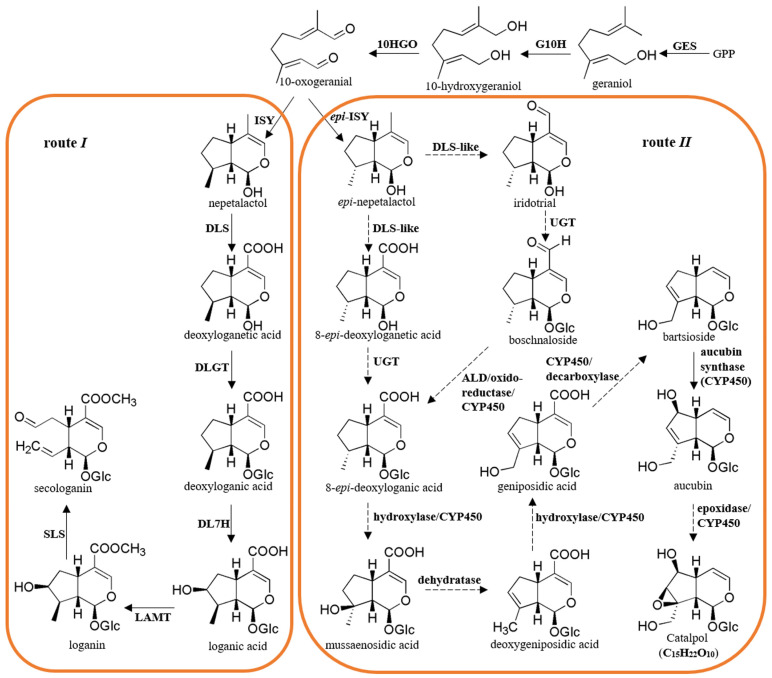
Proposed biosynthetic pathway of catalpol in *R. glutinosa.* GPP, geranyl diphosphate; GES, geraniol synthase; G10H, geraniol 10-hydroxylase; 10HGO, 10-hydroxygeraniol oxidoreductase; ISY, iridoid synthase; DLS, deoxyloganetic acid synthase; *epi*-ISY, *epi*-iridoid synthase; DLS-like, deoxyloganetic acid synthase-like; ALD, aldehyde dehydrogenase; DLGT, deoxyloganetic acid glucosyltransferase; DL7H, deoxyloganic acid 7-hydroxylase; LAMT, loganic acid methyltransferase; SLS, secologanin synthase; UGT, uridine diphosphate glycosyltransferase. The dotted lines indicate that the corresponding enzymes have not yet been isolated. The proposed pathway was prepared according to the previous studies and the KEGG PATHWAY Database [8,29].

Although catalpol displays a broad range of pharmaceutical activities, its biosynthesis mechanism remains obscure. As predicted in the literature, there are two principal routes for producing iridoids in plants. The biosynthetic *route I* can form iridoids of the 8β-series, such as seco-iridoids and their derivatives, while *route II* gives rise to the compounds of 8α-stereochemistry and *epi*-series, including catalpol, aucubin, and similar decarboxylated iridoid glucosides [8]. *Route I* has been well studied through feeding and molecular experiments. It has been reported that *route I* is initiated with the precursor geranyl diphosphate (GPP), which is generated by condensing isopentenyl diphosphate (IPP) and dimethylallyl diphosphate (DMAPP) from the mevalonate (MVA) and/or methylerythritol 4-phosphate (MEP) pathways. GPP is transferred to the monoterpene compound geraniol, then hydroxylated to produce 10-hydroxygeraniol and converted to 10-oxogeranial. The reduction and cyclization of 10-oxogeranial generate nepetalactol, and it is further modified by oxidation, glycosylation, hydroxylation, methylation, and a C-C bond cleavage reaction to produce series intermediates in sequence that are deoxyloganetic acid, deoxyloganic acid, loganic acid, loganin, and secologanin (Figure 1) [8]. Secologanin is considered the common precursor of seco-iridoids. The genes involved in this route were isolated from *Catharanthus roseus* and several other plants (Figure 1) [9,10,11,12,13,14,15,16,17,18,19].

*Route II* is identical to *route I* until 10-oxogeranial is generated. From 10-oxogeranial, *route II* splits into two branches, one of which produces 8-*epi*-deoxyloganic acid as follows: 10-oxogeranial→*epi*-nepetalactol→8-*epi*-deoxyloganetic acid→8-*epi*-deoxyloganic acid, which shares similar steps to *route I*, except for the different stereochemistry. The other branch involves 10-oxogeranial→*epi*-nepetalactol→*epi*-iridotrial→boschnaloside→8-*epi*-deoxyloganic acid (Figure 1) [20,21,22]. Through a sequence of reactions, including hydroxylation, dehydration, decarboxylation, and epoxidation, 8-*epi*-deoxyloganic acid is modified to form catalpol via a series of intermediates, including mussaenosidic acid, deoxygeniposidic acid, geniposidic acid, bartsioside, and aucubin (Figure 1) [23,24]. Based on our knowledge of the structures of these intermediates, the enzymes responsible for these steps from 10-oxogeranial to catalpol were proposed to be uridine diphosphate glycosyltransferase (UGT), aldehyde dehydrogenase (ALD), oxidoreductase, hydroxylase, dehydratase, decarboxylase, epoxidase, and cytochrome P450 (CYP450) (Figure 1). However, in addition to *epi*-iridoid synthase (*epi*-ISY) and aucubin synthase, no other gene in *route II* has been identified to date, and the molecular mechanism of catalpol biosynthesis remains unknown [25,26]. 

In recent years, comparative transcriptome analysis has been used as an efficient tool for exploring the biosynthesis of natural products. Zhi and Ma screened several enzymes, such as flavanone 3-dioxygenase, uroporphyrinogen decarboxylase, and squalene monooxygenase, which is probably involved in the downstream steps of catalpol biosynthesis through sequencing approaches [27,28]. However, other important tailoring enzymes, including CYP450, oxidoreductase, hydroxylase, dehydratase, decarboxylase, and epoxidase, have never been identified. To elucidate the biosynthesis of catalpol, young leaf transcriptomes were prepared from *R. glutinosa* Beijing No. 3, Huaifeng, and Jin No. 9 cultivars. The transcriptomes derived from the tuberous and adventitious roots of the Jin No. 9 cultivar were also constructed to narrow down the number of putative genes involved in catalpol formation. Using comparative transcriptome analysis, some candidate genes were identified in this study. To confirm the reliability of the gene screening results, the geraniol synthase, which was proposed to be the first committed enzyme in the formation of catalpol, was isolated and characterized to analyze its biochemical function. The results of this study enrich the gene information for elucidating the biosynthesis of catalpol in *R. glutinosa* and are beneficial for improving the medicinal quality of *R. glutinosa*.

## 2. Materials and Methods

### 2.1. Plant Materials

Three *R. glutinosa* cultivars, Beijing No. 3, Huaifeng, and Jin No. 9, were grown in the incubator at 23 ℃ under a 16 h–8 h light–dark cycle. An image of the *R. glutinosa* plant is shown in Supplementary Figure S1. The tuberous roots, adventitious roots, and young leaves were harvested 180 days after sprouting. The chemical standards catalpol and geraniol were purchased from Shanghai Source Leaf Biological Technology Company (Shanghai, China). GPP was from Sigma-Aldrich (Steinheim, Germany).

### 2.2. RNA Extraction and Transcriptome Construction

*R. glutinosa* samples (young leaves, tuberous roots, and adventitious roots) were collected and immediately ground to powder in liquid nitrogen. Total RNA was extracted using the Trizol reagent. The RNA quality and quantity were evaluated using Agilent 2100 Bioanalyzer (Agilent Technologies, Santa Clara, CA, USA) and Nanodrop spectrophotometer (Thermo Fisher Scientific, Waltham, MA, USA). The purity of RNA was analyzed using gel electrophoresis and Agilent 2100 Bioanalyzer. The Illumina NovaSeq 6000 platform (Illumina, San Diego, CA, USA) was applied to construct transcriptomes. The low-quality reads, including reads with adaptors, unknown nucleotides, or reads with more than 50% of bases with Q-value ≤20, were discarded to generate clean reads. Q20, Q30, and GC content of the clean data were calculated, and the downstream analyses were conducted based on the clean data with high quality. Transcriptome assembly was accomplished using Trinity software with min_kmer_cov set to 3 by default, and all other parameters set to default [30].

### 2.3. Functional Annotation

The function of unigenes was annotated based on several public databases, including the NCBI non-redundant protein (Nr, diamond v0.8.22, *e*-value = 10^−5^), the NCBI non-redundant nucleotide (Nt, blast 2.2.28, *e*-value = 10^−5^), Protein family (Pfam, HMMER 3.0 package, *e*-value = 10^−2^), the Swiss-Prot (diamond v0.8.22, *e*-value = 10^−5^), the Kyoto Encyclopedia of Genes and Genomes (KEGG, KAAS, *e*-value = 10^−10^), the euKaryotic Orthology Groups (KOG, diamond v0.8.22, *e*-value = 10^−3^), and Gene Ontology (GO, Blast2GO v2.5, *e*-value = 10^−6^). 

### 2.4. Identification of the Differentially Expressed Genes (DEGs)

The expression levels of unigenes were estimated by the fragments per kilo-base of the exon model per million mapped reads (FPKM) [31]. To identify the DEGs, the read counts were adjusted by an edgeR program package through one scaling normalized factor. Then, the DEGseq R package was used to perform differential expression analysis. *p* value was adjusted by q value, and q value < 0.005 with log2 (foldchange) > 1 was set as the threshold to identify significant DEGs [32]. GO enrichment analysis of these DEGs was accomplished using the GOseq R packages based on Wallenius non-central hyper-geometric distribution. KOBAS was applied to test the enrichment of DEGs in KEGG pathways [33].

### 2.5. HPLC Analysis

About 100 mg of ground plant materials of *R. glutinosa* was extracted with 1 mL of methanol in a bath sonicator for 1 h at room temperature. The methanol extract was evaporated to dryness and dissolved in 20% methanol. HPLC analysis was performed in a Shimadzu LC-16 machine with a UV detection system. An Inertsil ODS-SP reverse phase column (5 um, 250 mm × 4.6 mm) (Shimadzu, Kyoto, Japan) was used to separate different compounds with water (A) and acetonitrile (B) as the mobile phase in a stepped gradient mode as follows: 0.00–13.00 min, 3% B; 13.01–15.50 min, from 3 to 70% B; 15.51–20.00 min, 70% B; 20.01–25.00 min, 98% B; 25.01–35.00 min, 3% B. The flow rate was 1 mL/min with the column oven temperature set to 30 °C, and the detection wavelength was 210 nm. The standard catalpol curve was established to calculate the content of catalpol in different samples. 

### 2.6. Gene Expression Analysis

Gene expression level was analyzed by quantitative real-time PCR (qRT-PCR) based on the 2^−ΔΔCt^ method [34]. The *R. glutinosa TIP41* gene (GenBank accession no. KT306007) was chosen as an internal standard [35]. The gene-specific primers are listed in Supplementary Table S1. The qRT-PCR was performed with the Applied Biosystems QuantStudio5 Real-time PCR System using the FastStart Universal SYBR Green Mix (Roche) in three independent biological replicates with three technical replicates. The thermal cycling parameters were as follows: 95 °C for 10 min, followed by 40 cycles of 95 °C for 15 s, 60 °C for 1 min. 

### 2.7. Identification and Sequence Analysis of Candidate Geraniol Synthase Genes

To identify the putative geraniol synthase cDNAs (*RgGES*) from *R. glutinosa*, the published geraniol synthase from *C. roseus* (CrGES, GenBank accession No. JN882024.1) was used as the query sequence to perform a TBLASTN search against the *R. glutinosa* transcriptome. Multiple sequence alignment was conducted by the CLC Sequence Viewer 6.8 program and the phylogenetic tree was constructed using the Neighbor-Joining method in the MEGA 7.0 software.

### 2.8. Heterologous Expression of RgGES

The full-length coding sequence of the *RgGES* candidate was amplified with primer 27-28 and then subcloned into the expression vector pET28a at the *Bam*H I/*Sal* I site to generate the plasmid pET28a-*RgGES*. The expression of *RgGES* in *Escherichia coli* (*E. coli*) BL21(DE3) cells was induced with 0.3 mM isopropyl-β-D-thiogalactopyranoside (IPTG) at 16 °C, 160 rpm for 16 h. After the cultivation, the transgenic cells were collected by centrifugation and broken by ultrasonication in the lysis buffer (20 mM sodium phosphate, 300 mM sodium chloride with 10 mM imidazole; pH 7.5). The crude protein extracts were incubated with a HisPur Ni-NTA resin to purify the recombined RgGES protein following the manufacturer’s instructions. The purified RgGES protein was desalted into the assay buffer (100 mM HEPES-KOH, 1 mM MgCl_2_, pH 7.0). The concentration of RgGES protein was evaluated by the Nanodrop spectrophotometer and its purity was analyzed using SDS-PAGE.

### 2.9. Enzyme Assays

Enzyme assay was performed in 800 μL of 100 mM HEPES-KOH buffer (pH 7.0 containing 1 mM MgCl_2_), 10 mM GPP, and 3 μg of the purified RgGES protein. The reaction mixture was overlaid with 800 μL of hexane and incubated at 30 °C for 2 h. By thoroughly vortexing for 5 min, the reaction was stopped. Then, the hexane phase was collected by centrifugation and analyzed through GC-MS. The empty vector pET28a was used as a control. Enzyme assay was performed according to the previous literature with minor modifications [36]. 

### 2.10. GC-MS Analysis

GC-MS analyses were carried out on the 8890 gas chromatograph coupled to a 7000D mass spectrometer (Agilent Technologies, Santa Clara, CA, USA). The products were analyzed using an HP-5 MS column (30 m × 0.25 mm × 0.25 μm film thickness) with the carrier gas being helium at a flow rate of 1 mL/min. For analyzing the enzymatic products, the initial oven temperature was set at 60 °C for 1 min, followed by a 5 °C/min ramp to 150 °C, held at 150 °C for 5 min, then followed by a linear gradient to 240 °C at a rate of 20 °C/min, and held for 3 min. The splitless injection mode was used and the injector temperature was 230 °C. Full mass spectra were generated by scanning within the *m*/*z* range of 40–500 u. The compounds were confirmed by comparing the retention time and mass fragmentation patterns with those of authentic standards. The GC-MS detection method was determined based on the previous study [37].

The methodology in this study was summarized as a flowchart in Supplementary Figure S2.

## 3. Results

### 3.1. HPLC Analysis of Catalpol in R. glutinosa 

**Figure 2:**
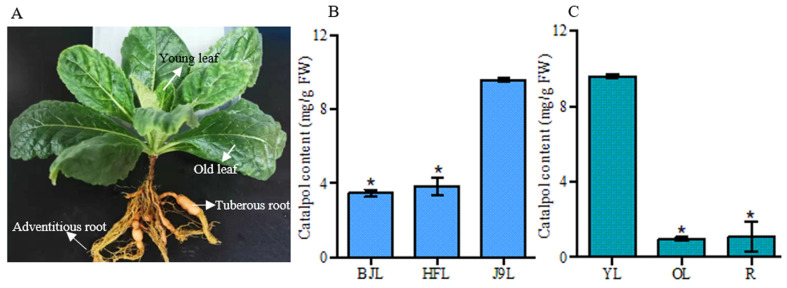
(**A**) *R. glutinosa* plant; (**B**) the content of catalpol in the young leaves of different *R. glutinosa* cultivars. BJL, young leaves of Beijing No. 3; HFL, young leaves of Huaifeng; J9L, young leaves of Jin No. 9; (**C**) the concentration of catalpol in different parts of *R. glutinosa*. YL, young leaf; OL, old leaf; R, tuberous root. Error bars represent the standard errors of the means (SEMs) calculated from three biological replicates. Asterisks indicate a significant difference (*p* < 0.05).

Considering that catalpol was the major active compound of *R. glutinosa*, its production in the Beijing No. 3, Huaifeng, and Jin No. 9 cultivars was analyzed. The result showed that the content of catalpol was higher in the young leaves of the Jin No. 9 cultivar than that of the Beijing No. 3 and Huaifeng cultivars (Figure 2B). Then, different parts of the *R. glutinosa* Jin No. 9 cultivar, including the young leaves, old leaves, tuberous roots, and adventitious roots were selected to explore the catalpol distribution pattern. As shown in Figure 2C, catalpol was synthesized at a higher level in the young leaves than in the old leaves and tuberous roots. The content of catalpol in the old leaves was comparable with that in the tuberous roots. Notably, catalpol was not detected in the adventitious roots.

### 3.2. Transcriptome Sequencing and De Novo Assembly

To globally identify candidate genes in the biosynthesis of valuable compounds in *R. glutinosa*, transcriptomes from the young leaves, tuberous roots, and adventitious roots of the Jin No. 9 cultivar, as well as the young leaves of the Beijing No. 3 and Huaifeng cultivars, were constructed using an Illumina NovaSeq 6000 platform, and were designated as J9L, J9R, J9AR, BJL, and HFL, respectively. As a result, more than 29 million clean reads per transcriptome were produced. The Q20 and Q30 of all the libraries were over 97.75% and 93.19%, indicating these clean data could be used for further analysis (Table 1). The error rate of all the libraries was 0.03, and its distribution is provided in Supplementary Figure S3. The clean reads from all the transcriptomes were assembled into 71,142 unigenes with a mean length of 1131 bp and an N50 length of 1732 bp (Supplementary Table S2). The raw data from the transcriptomes were submitted to the publicly accessible Genome Sequence Archive (GSA) database in the BIG Data Center under the accession number CRA005581.

### 3.3. Functional Annotation and Classification of the R. glutinosa Unigenes

A total of 71,142 unigenes were successfully annotated by searching all these sequences against seven public databases, including the Nr, Nt, Pfam, Swiss-Prot, KEGG, KOG, and GO. Among these databases, the highest annotation percentage was in the Nr (38,788 unigenes; 54.52%), followed by the Nt database (30,861 unigenes; 43.37%) and the Swiss-Prot (29,731 unigenes; 41.79%) (Table 2).

**Figure 3:**
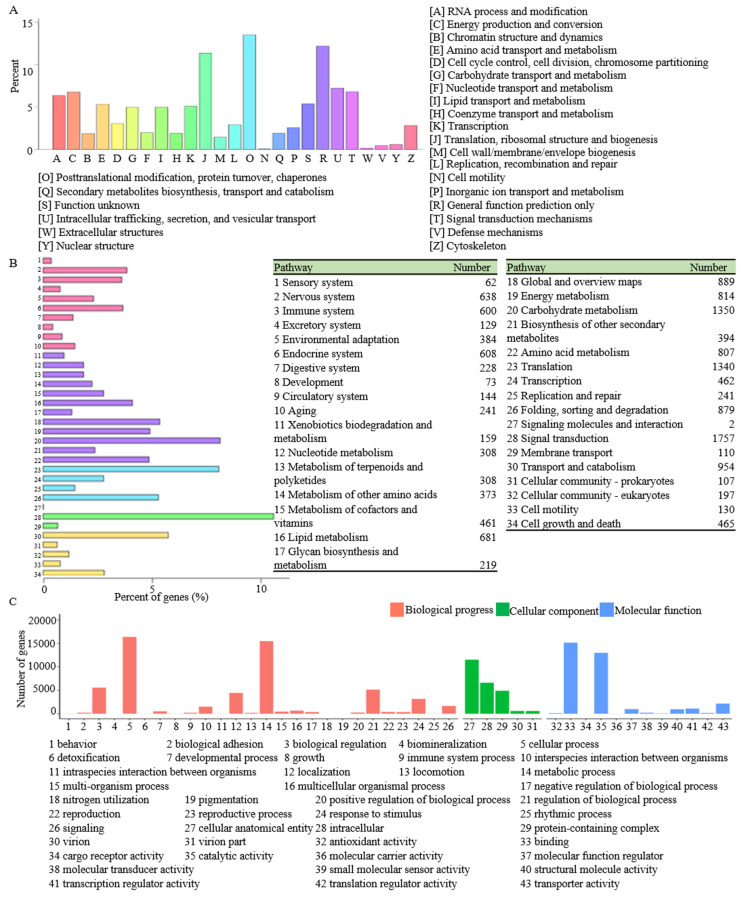
KOG annotation (**A**), KEGG classification (**B**), and GO classification (**C**) of all unigenes.

The KOG annotation revealed that 10,119 unigenes were classified into 25 terms, of which the largest category was “posttranslational modification, protein turnover, chaperones”, including 1367 unigenes, followed by “general function prediction only” (1232 unigenes) and “translation, ribosomal structure, and biogenesis” (1150 unigenes) (Figure 3A, Supplementary Table S3). To further explain the function of unigenes in biological pathways, all the unigenes were searched against the KEGG database. The result showed 13,862 unigenes were classified into 34 KEGG pathways (Figure 3B, Supplementary Table S4), of which “signal transduction” (1757 unigenes), “carbohydrate metabolism” (1350 unigenes), and “translation” (1340 unigenes) were the highest represented pathways. To functionally classify the *R. glutinosa* transcriptome, 28,511 unigenes were categorized into three GO terms: biological process, cellular component, and molecular function. The subcategory for “cellular process” (16,395 unigenes) was the most abundant group, followed by “metabolic process” (15,485 unigenes) and “binding” (15,155 unigenes) (Figure 3C, Supplementary Table S5).

**Figure 4:**
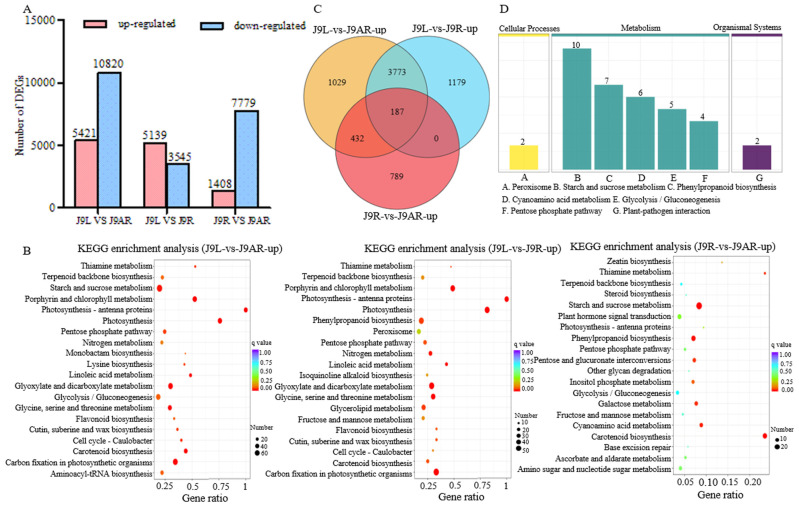
Number of DEGs (**A**) and KEGG enrichment analysis of up-regulated genes (**B**) between different parts of *R. glutinosa*; (**C**) Venn diagram of up-regulated unigenes in different tissues of *R. glutinosa* (J9L vs. J9AR, J9L vs. J9R, and J9R vs. J9AR); (**D**) representative pathway of the common up-regulated genes from J9L vs. J9AR, J9L vs. J9R, and J9R vs. J9AR.

The FPKM method was applied to calculate the transcript abundance of the unigenes. Given that the content of catalpol is highest in the young leaves, followed by the tuberous roots and adventitious roots, the DEGs between the different tissues of *R. glutinosa* were screened. A total of 16,241 DGEs were obtained from the J9L library versus the J9AR library, including 5421 up-regulated DEGs and 10,820 down-regulated DEGs (Figure 4A). The GO annotation of these up-regulated genes showed that the highest number of unigenes was under the term of “catalytic activity” (1662 unigenes), followed by “single-organism metabolic process” (886 unigenes). For the up-regulated genes in the J9L library, the top three enriched pathways were “starch and sucrose metabolism”, “carbon fixation in photosynthetic organisms”, and “glyoxylate and dicarboxylate metabolism” (Figure 4B). By comparing the J9L library with the J9R library, 5139 up-regulated DGEs as well as 3545 down-regulated DGEs were identified. The up-regulated genes were mainly classified into the pathway “carbon fixation in photosynthetic organisms” (Figure 4B). When the J9R library was compared to the J9AR library, 1408 up-regulated genes and 7779 down-regulated ones were generated, and the enriched pathway of the up-regulated genes in the J9R library was “starch and sucrose metabolism” (Figure 4B). A total of 187 unigenes showed up-regulation in all the comparisons, including the J9L library versus the J9AR library, the J9L library versus the J9R library, and the J9R library versus the J9AR library, exhibiting positive correlation with the content of catalpol (Figure 4C). The top three representative pathways of these 187 sequences were “starch and sucrose metabolism”, “phenylpropanoid biosynthesis”, and “cyanoamino acid metabolism” (Figure 4D).

### 3.4. Identification of Putative Genes in the Pathway of Catalpol Biosynthesis

**Figure 5:**
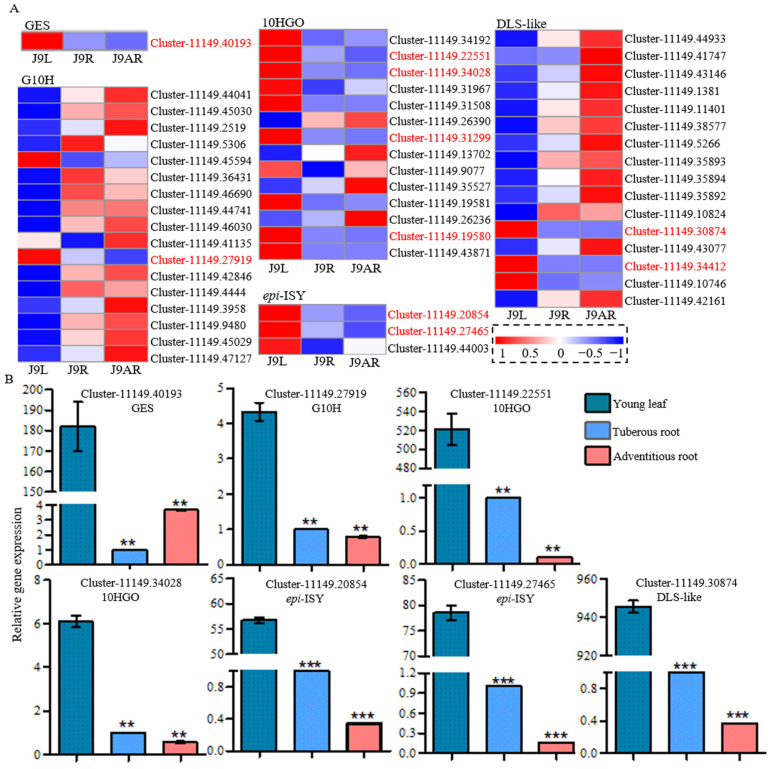
Heatmap based on the FPKM data (**A**) and qRT-PCR analysis (**B**) of the putative common genes in route *II* from different parts of *R. glutinosa*. Asterisks indicate a significant difference (** *p* < 0.01; *** *p* < 0.001).

We focused attention on the downstream steps to catalpol. According to the proposed biosynthetic pathway of catalpol shown in Figure 1, GPP was converted to 10-oxogeranial by GES, G10H, and 10HGO in sequence, which were common enzymes in *route I* and *route II*. Subsequently, *epi*-nepetalactol was formed from 10-oxogeranial by *epi*-ISY protein, followed by its conversion into either 8-*epi*-deoxyloganetic acid or iridotrial by DLS-like enzyme. The candidate genes for these enzymes were screened by keyword searching, and a total of 51 sequences were found (Supplementary Table S6). Among them, several unigenes, including one for GES (Cluster-11149.40193), one for G10H (Cluster-11149.27919), four for 10HGO (Cluster11149.19580, Cluster-11149.22551, Cluster-11149.34028, and Cluster-11149.31299), two for *epi*-ISY (Cluster-11149.20854 and Cluster-11149.27465), and two for DLS-like (Cluster-11149.30874 and Cluster-11149.34412), were selected as putative genes involved in catalpol biosynthesis, due to their similar expression to the accumulation pattern of catalpol. These putative candidate genes displayed their presence in all the Beijing No. 3, Huaifeng, and Jin No. 9 cultivars; meanwhile, their highest expression level was in the young leaves, followed by the tuberous roots and adventitious roots (Figure 5A). Discarding two unigenes for 10HGO (Cluster-11149.31299 and Cluster-11149.19580) and one for DLS-like (Cluster-11149.34412), the others with complete open reading frames (ORFs) were selected for the qRT-PCR analysis. The expression levels of these unigenes were consistent between the qRT-PCR results and the FPKM data, except for Cluster-11149.40193 (Figure 5B). The qRT-PCR melting curves are shown in Supplementary Figure S4. Additionally, GES (AFD64744.1), G10H (CAC80883.1), 10HGO (AHA82031.1), ISY (AFW98981.1), and DLS (AHX24370.1) from *C. roseus* were used as the query sequences to conduct the tBLASTn searching, and the highest hits in the *R. glutinosa* transcriptomes were Cluster-11149.40193 (GES), Cluster-11149.27919 (G10H), Cluster-11149.22551 (10HGO), Cluster-11149.27465 (*epi*-ISY), and Cluster-11149.11401 (DLS-like), respectively (Supplementary Table S6).

**Figure 6:**
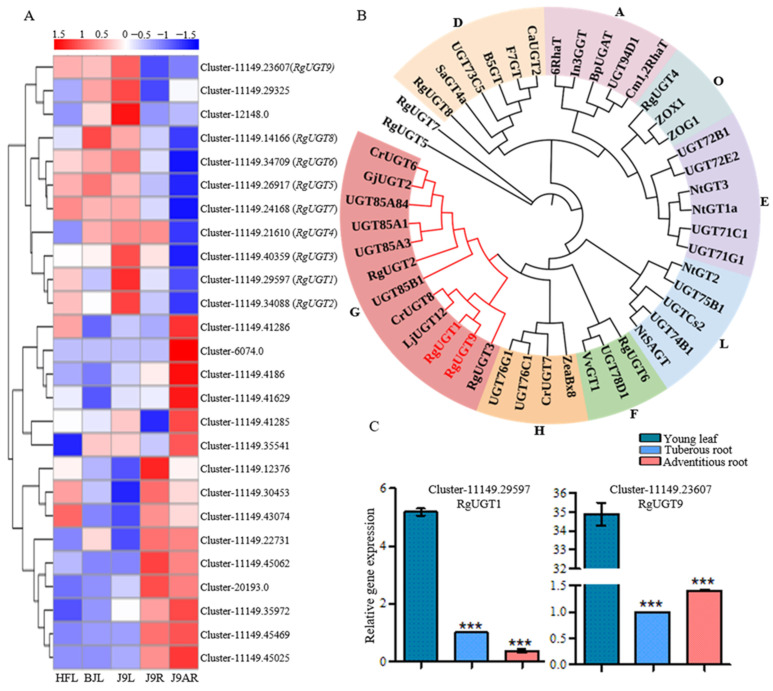
(**A**) The heatmap showing an expression clustering of putative UGT genes in catalpol biosynthesis; (**B**) phylogenetic analysis of RgUGT1-9 with other known UGTs from different groups. The tree was constructed with the Neighbor-Joining method (with 1000 bootstrap replications) in the MEGA 7.0 software. Accession numbers of UGTs were shown in Supplementary Table S8; (**C**) the qRT-PCR analysis of UGT candidate genes in catalpol formation. Asterisks indicate a significant difference (*** *p* < 0.001).

As depicted in Figure 1, UGT catalyzed the conversion of 8-*epi*-deoxyloganetic acid and iridotrial into 8-*epi*-deoxyloganic acid and boschnaloside, respectively. A total of 167 UGT unigenes were discovered in the *R. glutinosa* transcriptomes (Supplementary Table S7) while 8 of them (RgUGT1-8) satisfied our gene screening criteria. In addition, 21 sequences were annotated as UGT candidates related to the iridoid glucosides biosynthesis. The cluster analysis of all these UGT candidate genes revealed that RgUGT1-8 were assigned to the same cluster, which formed a separate group with other UGT candidates (Figure 6A). CrUGT8 (AB733667) from *C. roseus*, which glycosylated deoxyloganetic acid to deoxyloganic acid in *route I*, was used as the query sequence to search in the *R. glutinosa* transcriptomes. As a result, the highest hit was Cluster-11149.23607 (RgUGT9), which was highly expressed in the adventitious roots compared to the tuberous roots [10]. Previous studies have suggested that UGT genes involved in the production of iridoids usually belong to group G of the Family 1 plant secondary product glycosyltransferases (PSPGs). The members of Family 1 PSPGs are responsible for the glycosylation of various natural plant products [10]. The phylogenetic analysis of RgUGT1-9 showed that RgUGT1,2,3,9 fell into group G, while RgUGT4 and RgUGT6 belonged to group O and F, respectively. RgUGT1 and RgUGT9 were closely related to CrUGT8 (Figure 6B, Supplementary Table S8). Furthermore, the qRT-PCR analysis revealed that RgUGT1 and RgUGT9 were highly expressed in the young leaves compared to the roots (Figure 6C).

It has been predicted that ALDs/NADP^+^ oxidoreductases might catalyze the oxidation of boschnaloside to form 8-*epi*-deoxyloganic acid. In the transcriptomic databases, 104 genes for aldehyde dehydrogenases and 439 genes for NADP^+^ oxidoreductases were identified. Among them, 13 aldehyde dehydrogenase genes and 70 NADP^+^ oxidoreductase genes met the gene screening criteria and showed significantly higher expression levels in the young leaves than those in the tuberous and adventitious roots (Supplementary Tables S9 and 10).

The cytochrome P450 enzymes (CYP450s) exhibit a broad range of activities in hydroxylation, epoxidation, decarboxylation, as well as C-C oxidative cleavage reactions. CYP450s might participate in a series of modifications in catalpol biosynthesis (Figure 1) [38]. A total of 363 unigenes were annotated as CYP450s while the expression level of 44 genes correlated well with the catalpol content in *R. glutinosa* (Supplementary Table S11). Given that the reported CYP450 genes, including G10H, DLS, DL7H, and SLS in *route I* of iridoid biosynthesis, belong to the CYP72 and CYP76 families, these 44 candidate sequences were further screened, resulting in 12 CYP72 and 7 CYP76 sequences, respectively (Supplementary Table S11). Among these CYP450 members, Cluster-11149.22060 showed the highest sequence similarity with the reported aucubin synthase [26], which exactly met the gene screening criteria of this study (Supplementary Table S11). 

**Figure 7:**
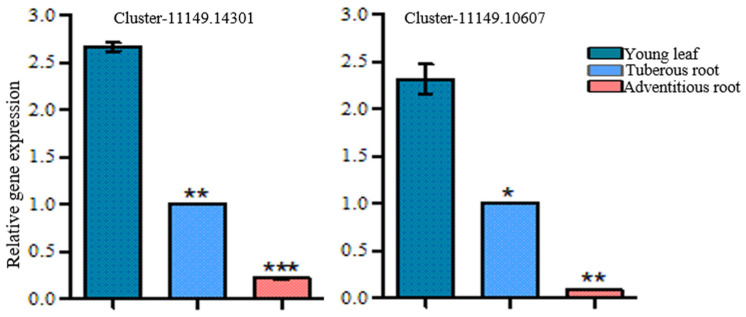
qRT-PCR analysis of hydroxylase candidate genes in *R. glutinosa*. Asterisks indicate a significant difference (* *p* < 0.05; ** *p* < 0.01; *** *p* < 0.001).

The dehydration of mussaenosidic acid may be catalyzed by dehydratase. In the *R. glutinosa* transcriptomes, there were 85 sequences annotated as dehydratase, of which 22 candidates showed higher expression levels in the young leaves compared with the roots, and met the screening criteria (Supplementary Table S12). The transformation of geniposidic acid to bartsioside can take place by decarboxylation, which is usually catalyzed by decarboxylase. By keyword searching, a total of 239 decarboxylase unigenes were found, and the expression level of 30 sequences matched the accumulation pattern of catalpol (Supplementary Table S13). Hydroxylase and epoxidase also participate in the biosynthesis of catalpol. Hydroxylase is proposed to catalyze the hydroxylation of 8-*epi*-deoxyloganic acid, deoxygeniposidic acid, and bartsioside, while epoxidase can convert aucubin to form catalpol. A total of 19 genes for hydroxylases and 10 genes for epoxidases, which met the gene screening criteria herein, were predicted to be involved in catalpol formation in *R. glutinosa* (Supplementary Table S14). Among these hydroxylase candidates, Cluster-11149.14301 and Cluster-11149.10607 were annotated as iridoid hydroxylase and exhibited higher expression levels in the young leaves than in the roots (Figure 7). The heatmap of all the candidate genes in the biosynthetic pathway of catalpol is shown in Supplementary Figure S5.

### 3.5. Cloning and Sequence Analysis of RgGES in R. glutinosa

Given that geraniol synthase plays an important role in the production of catalpol, it is necessary to identify the corresponding gene from *R. glutinosa* and analyze its function. Cluster-11149.40193, which was annotated as *GES*, was selected for further analysis. As a result, two homologous *GES* genes (*RgGES1* and *RgGES2*) with several mutations were obtained by the PCR method (Supplementary Figure S6). The ORFs of *RgGES1* (GenBase Accession No. C_AA047852.1) and *RgGES2* (GenBase Accession No. C_AA047853.1) were 1749 bp, which encoded 582 amino acids. 

**Figure 8:**
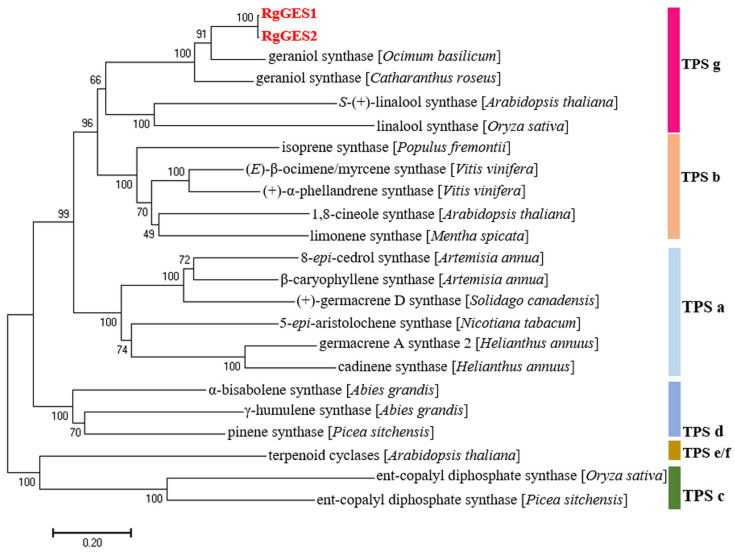
Phylogenetic analysis of the putative RgGESs with other known terpene synthases from different subfamilies. The tree was constructed using the Neighbor-Joining method in the MEGA 7.0. TPS a–g stand for terpene synthase subfamilies. Accession numbers of various TPSs are provided in Supplementary Table S15.

**Figure 9:**
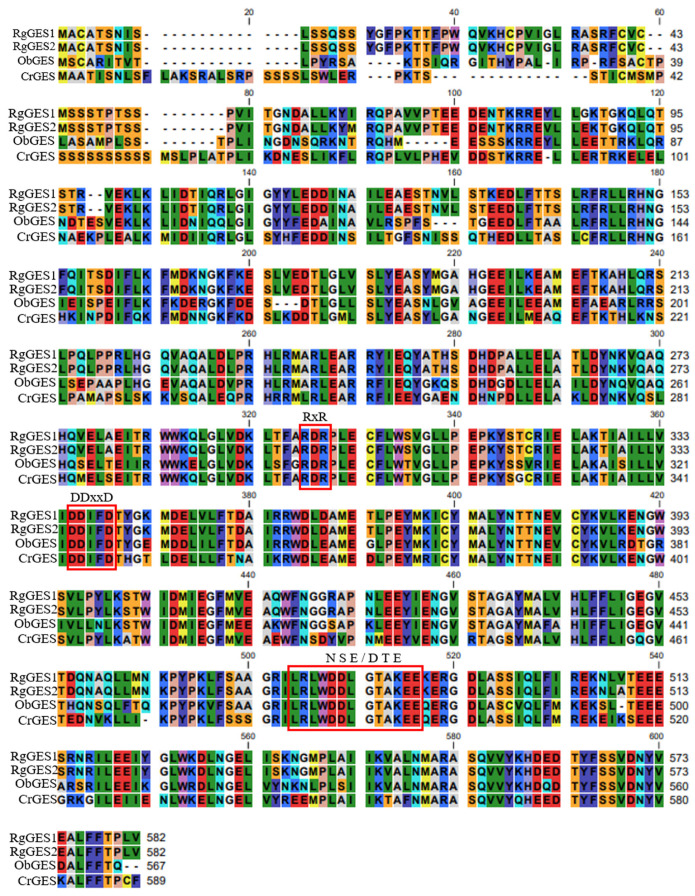
Amino acid sequence alignment of RgGESs and other GESs. The CLC Sequence Viewer program was used to conduct the multiple sequence alignment. Highly conserved motifs of TPSs were indicated with boxes, including DDxxD, RxR, and NSE/DTE. ObGES, *O. basilicum*, AAR11765.1; CrGES, *C. roseus*, AFD64744.1.

The phylogenetic analysis of the RgGESs with the known terpene synthases from other plants revealed that RgGES1 and RgGES2 were classified into the TPS g group [39], and the RgGES proteins were most closely related to the geraniol synthase from *Ocimum basilicum* (Figure 8). The multiple sequence alignment showed that RgGES1 and RgGES2 contained several highly conserved domains, similar to the GESs from *C. roseus* (AFD64744.1) and *O. basilicum* (AAR11765.1), including DDxxD, RxR, and NSE/DTE (Figure 9). The DDxxD and NSE/DTE motifs are important for the fixation of the pyrophosphate substrate, while RxR, which is located about 35 amino acids ahead of the DDxxD motif, is involved in the complexation of the diphosphate [40]. 

### 3.6. Functional Characterization of RgGES Genes 

**Figure 10:**
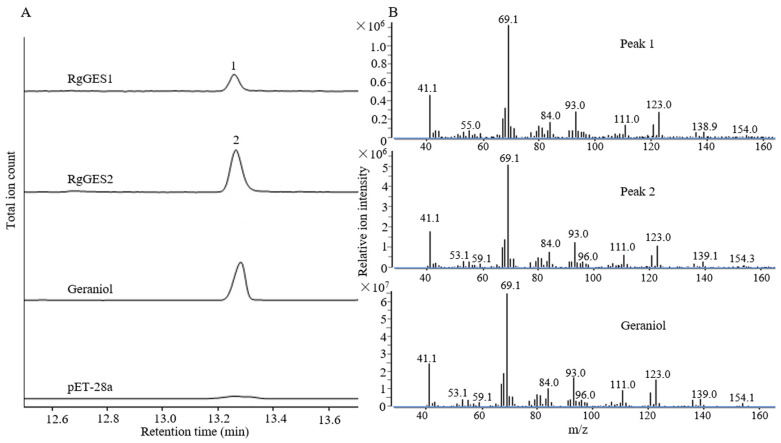
GC-MS analysis of the products in the in vitro assays using the purified RgGESs proteins (**A**) and mass spectra of the peaks from A (**B**). The protein extracted from the *E. coli* cells harboring the empty vector pET28a was used for the control reaction. Total ion chromatograms were shown for the reaction with RgGES1 and RgGES2 yielding geraniol (peaks 1 and 2).

To verify the biochemical activity of the RgGESs from *R. glutinosa*, *RgGES1* and *RgGES2* were subcloned into a pET28a plasmid to construct pET28a-*RgGES1*-*2* vectors, and subsequently expressed in *E. coli* BL21(DE3) cells. The recombined RgGES proteins were purified using a His-tag (Supplementary Figure S7) and further incubated with the substrate GPP. The enzymatic products were detected by GC-MS. In comparison with the control reaction, both RgGES1 and RgGES2 catalyzed GPP to produce a new peak (Figure 10), which showed identical retention time and mass fragmentation patterns to those of the authentic standard geraniol, suggesting RgGES1 and RgGES2 converted GPP to geraniol.

## 4. Discussion

*R. glutinosa* has been extensively utilized as a medicinal plant to clear away *heat* and promote *salivation* [1]. Catalpol, the main active constituent in *R. glutinosa*, has been reported to possess anti-inflammatory, anti-oxidant, anti-diabetic, and neuroprotective effects. Previous studies have investigated catalpol accumulations, and found that different cultivars of *R. glutinosa*, as well as distinct plant samples from the same cultivar, showed diverse catalpol production abilities [41]. Ji discovered a correlation between catalpol concentration and growth stage [42]. Furthermore, our unpublished data indicated that the difference in the accumulation of catalpol existed among different leaves from the same plant. Therefore, it is necessary to analyze the catalpol content before constructing *R. glutinosa* transcriptomes. Herein, we employed HPLC to detect the content of catalpol in different cultivars and tissues. Under the condition of this study, young leaves of the Jin No. 9 cultivar exhibited a higher catalpol accumulation than that of the Beijing No. 3 and Huaifeng cultivars (Figure 2). The HPLC analysis of the different tissues of the Jin No. 9 cultivar revealed that the concentration of catalpol was significantly higher in the young leaves compared with the other parts, suggesting the young leaves were more suitable for isolating catalpol. This result was consistent with Ji’s study [42]. 

Transcriptome analysis has been a universal tool to investigate molecular mechanisms. In the case of *R. glutinosa*, Li and Sun employed transcriptome sequencing technology to identify miRNAs and genes associated with replanting disease and root development, respectively [43,44]. RNA sequencing technology was also applied to discover functional genes involved in natural product formation in *R. glutinosa*. To explore the biosynthesis of acteoside in *R. glutinosa*, Zhou and Wang established transcriptomic databases from the tuberous roots and the hairy roots, respectively [45,46]. Sun constructed an EST dataset from the roots and subsequently screened a number of genes in the MVA and MEP pathways, as well as GES, G10H, and 10HGO in the upstream pathway of iridoid biosynthesis [47]. In addition, Zhi constructed transcriptome libraries from the radial striation and non-radial striation of tuberous roots to identify several enzymes in the downstream pathway of catalpol production, such as iridoid synthase, cytochrome P450 monooxygenase, aldehyde dehydrogenase, flavanone 3-dioxygenase, uroporphyrinogen decarboxylase, and squalene monooxygenase [27]. Ma reported genome information of *R. glutinosa* using nanopore technology. Besides the enzymes mentioned in Zhi’s research, Ma also identified DL7H, DLGT, LAMT, and SLS in *route I* of iridoid biosynthesis [28]. Zhou constructed the metabolome and transcriptome from *R. glutinosa* dedifferentiated cells and cambial meristematic cells, and further identified neomenthol dehydrogenase, cytochrome P450 family 76 subfamily A, 7-deoxyloganetin glucosyltransferase, and 8-carboxylinalool synthase in the downstream steps of monoterpenoid biosynthesis [48]. Although previous studies have identified some enzyme-coding genes in catalpol formation, there are still a number of important enzymes that have never been reported in *R. glutinosa*, such as CYP450, oxidoreductase, dehydratase, hydroxylase, and epoxidase. These enzymes probably participate in modifying the *epi*-nepetalactol skeleton to catalpol. 

Given the fact that catalpol is highly accumulated in the young leaves, we constructed transcriptomes from the young leaves of three *R. glutinosa* cultivars (Beijing No. 3, Huaifeng, and Jin No. 9 cultivars) to elucidate the catalpol biosynthetic pathway in this study. Moreover, considering the difference in the catalpol content among different tissues, the transcriptomic data from the tuberous and adventitious roots were further obtained. The transcriptomes yielded a total of 71,142 unigenes with functional annotation. The number of functionally annotated unigenes was more than that in the tuberous root transcriptome from the *R. glutinosa* Wen 85-5 cultivar, but less than that generated from root databases from 1706, BJ1, Wen 85-5, and QH1 cultivars [27,45]. This difference might result from the use of different cultivars and tissues in different studies. The transcriptome from the adventitious roots which had no catalpol was first applied to reduce the number of candidate genes in catalpol biosynthesis. The adventitious root library can provide more reliable evidence for the comparative transcriptome analysis. According to the HPLC detection results, the rationale for discovering genes in the catalpol biosynthetic pathway was designed as follows: first, compared with the adventitious roots, candidate genes would show higher expression in the young leaves with fold changes larger than two, and second, the transcript level of the selected genes would be highest in the young leaves, followed by the tuberous roots and the adventitious roots, in sequence. In addition, the candidate genes would be present in all the Beijing No. 3, Huaifeng, and Jin No. 9 cultivars. Using the comparative transcriptome analysis, more candidate genes in the downstream pathway of catalpol biosynthesis were obtained in this study. 

As a typical iridoid glucoside with 8α-stereochemistry, catalpol may be formed by *route II*: geraniol→10-hydroxygeraniol→10-oxogeranial→*epi*-nepetalactol→8-*epi*-deoxyloganetic acid or boschnaloside→8-*epi*-deoxyloganic acid. 8-*epi*-deoxyloganic acid is subsequently modified to catalpol by hydroxylation, dehydration, decarboxylation, and epoxidation (Figure 1) [8,22,24]. However, the knowledge of genes responsible for the biosynthetic steps in *route II* is poor. In this study, the formation of catalpol is divided into two periods to conduct analysis. The first period is the production of 8-*epi*-deoxyloganic acid from GPP, which shares similar steps with *route I*. The first step of this period is the conversion of GPP to geraniol by GES. A GES candidate gene (Cluster-11149.40193) was found in *R. glutinosa*, and it showed 71% amino acid identity with the GES from *C. roseus.* It is predicted that the transformation of geraniol to 8-*epi*-deoxyloganic acid is catalyzed by common enzymes, such as G10H, 10HGO, *epi*-ISY, and DLS-like. Using keyword and blast searching approaches, all these common enzymes were found here. Nine sequences encoding these four enzymes met the gene screening criteria in this study. These candidates showed identical expression features to the accumulation pattern of catalpol (Figure 5), indicating their possible roles in catalpol production. Glycosylation reactions in secondary metabolism are usually catalyzed by glycosyltransferases from Family 1 PSPGs [10]. A total of 167 UGT sequences were identified, and the transcript level of eight unigenes (RgUGT1-8) matched the catalpol accumulation. However, the other UTG sequence Cluster-11149.23607 (RgUGT9) showed the highest amino acid identity with CrUGT8, a reported UGT in iridoid biosynthesis. The phylogenetic analysis suggested that RgUGT1,2,3,9 belonged to group G (Figure 6), which contains several iridoid-specific glucosyltransferase members, indicating these unigenes might play roles in the catalpol biosynthesis in *R. glutinosa.* Considering that sequence homology is not enough to predict the precise enzymatic activity of glycosyltransferase, RgUGT1-3 as well as RgUGT9 will be further cloned to analyze their functions. 

The conversion of 8-*epi*-deoxyloganic acid to catalpol is considered the second period of catalpol production. Cytochrome P450s play important roles in this stage. A total of 44 unigenes of the CYP450 family met the screening criteria based on the FPKM analysis. Given that the known CYP450s in *route I* belong to the CYP72 and 76 families, 12 unigenes of the CYP72 family and 7 members of the CYP76 family were further identified in this study. The top three most highly expressed sequences of the CYP72 family were annotated as dehydrogenase, oxidoreductase, and SLS, respectively. The highest expressed unigenes of the CYP76 family were DLS and G10H. The identification of genes in the iridoids biosynthesis suggested that the keyword searching method and the gene screening criteria were reliable in discovering putative genes in catalpol biosynthesis. As shown in Figure 1, hydroxylases are responsible for three steps in catalpol formation. The step from 8-*epi*-deoxyloganic acid to mussaenosidic acid is similar to the reaction catalyzed by DL7H in *route I*. Herein, two unigenes (Cluster-11149.14301 and Cluster-11149.10607) were annotated as DL7H, and their expression profile showed a positive correlation with the accumulation of catalpol (Figure 7), suggesting these two unigenes might catalyze the hydroxylation of 8-*epi*-deoxyloganic acid. 

Geraniol synthase is the first branch enzyme in the biogenesis of catalpol. In the *R. glutinosa* transcriptomic databases, Cluster-11149.40193 was selected as a *GES* candidate for functional characterization. Using the PCR approach, *RgGES1* and *RgGES2* with several mutations were isolated from the young leaves. Since *R. glutinosa* is an autotetraploid plant [28], it is reasonable to isolate two similar sequences from *R. glutinosa* for one unigene. The phylogenetic analysis revealed RgGES1 and RgGES2 belonged to the TPS g group, which specifically produce acyclic terpenes like geraniol and linalool. As the general structural feature of the members from the TPS g group, RgGES1 and RgGES2 lacked the RRX_8_W motif, which has been present in many, but not always found in, monoterpene synthases [49]. Like the geraniol synthase from *O. basilicum* and *Dendrobium officinale* [32,50], the purified recombinant RgGES1 and RgGES2 catalyzed GPP to uniquely produce geraniol in the in vitro assay (Figure 10), suggesting RgGES1 and RgGES2 were classified as geraniol synthase in *R. glutinosa*. In addition, given that *RgGES* (Cluster-11149.40193) is highly expressed in the young leaves, it was reasonable to assume that *RgGES1* and *RgGES2* participate in catalpol biosynthesis in *R. glutinosa*.

## 5. Conclusions

Transcriptome sequencing was performed in the young leaves of *R. glutinosa* Beijing No. 3, Huaifeng, and Jin No. 9 cultivars. Meanwhile, transcriptomes from the tuberous and adventitious roots of the Jin No. 9 cultivar were also constructed. Using the comparative transcriptome analysis, many important candidate genes that participated in catalpol biosynthesis were identified, such as CYP450, UGTs, oxidoreductases, hydroxylase, and decarboxylases. In addition, *RgGES1* and *RgGES2* were isolated and characterized as geraniol synthase, which catalyzed GPP to produce the precursor geraniol. The findings in this study will facilitate functional studies of catalpol biogenesis in *R. glutinosa*.

## Figures and Tables

**Table 1:** Statistics of sequencing of *R. glutinosa* transcriptomes.

Sample	Raw Reads	Clean Reads	Clean Bases	Error Rate	Q20 (%)	Q30 (%)	GC (%)
J9L	31,180,906	29,195,338	8.76 G	0.03	97.84	93.43	45.42
J9R	31,674,784	30,297,505	9.09 G	0.03	97.85	93.38	44.44
J9AR	30,827,652	29,371,270	8.81 G	0.03	97.75	93.19	45.10
BJL	30,882,434	29,153,263	8.75 G	0.03	97.83	93.36	45.02
HFL	31,220,770	29,902,124	8.97 G	0.03	98.01	93.91	44.47

**Table 2:** Statistics of annotation for assembled unigenes from *R. glutinosa*.

Databases	Number of Unigenes	Percentage
Annotated in all databases	6958	9.78
Annotated in KOG	10,119	14.22
Annotated in KEGG	13,862	19.48
Annotated in GO	28,511	40.07
Annotated in Pfam	28,512	40.07
Annotated in Swiss-Prot	29,731	41.79
Annotated in Nt	30,861	43.37
Annotated in Nr	38,788	54.52
Annotated in at least one database	71,142	100
Total unigenes	71,142	100

## Data Availability

The transcriptome data have been deposited in the publicly accessible Genome Sequence Archive (GSA) database in the BIG Data Center under the accession number CRA005581. *RgGES1* and *RgGES2* were submitted to the GenBase database in the BIG Data Center under the accession number C_AA047852.1 and C_AA047853.1, respectively.

## Supplementary Materials

The following supporting information can be downloaded at https://www.mdpi.com/article/10.3390/genes15020155/s1, Supplementary Figure S1: The *R. glutinosa* plant; Supplementary Figure S2: The flowchart to the Material and Method section for summarizing methodology; Supplementary Figure S3: The error rate distribution of unigenes; Supplementary Figure S4: The melting curves of the genes chosen for qRT-PCR analysis; Supplementary Figure S5: The heatmap of differentially expressed genes that were evaluated in this study; Supplementary Figure S6: Agarose gel electrophoresis for PCR products of *RgGES*; Supplementary Figure S7: The SDS-PAGE analysis of the recombinant RgGES1 and RgGES2 protein; Supplementary Table S1: Primers used in this study; Supplementary Table S2: Transcriptome assembly statistics for *R. glutinosa*; Supplementary Table S3: Annotation of unigenes in KOG database; Supplementary Table S4: KEGG classification of all the unigenes; Supplementary Table S5: Summary of annotated unigenes in GO database; Supplementary Table S6: Candidate unigenes of common enzymes in *route I* and *route II*; Supplementary Table S7: Candidate UGT unigenes in *R. glutinosa* transcriptomes; Supplementary Table S8: Accession numbers of PSPGs used for phylogenetic analysis in Figure 6; Supplementary Table S9: Aldehyde dehydrogenase unigenes identified in *R. glutinosa* transcriptomes; Supplementary Table S10: NADP^+^ oxidoreductase candidate genes in *R. glutinosa* transcriptomes; Supplementary Table S11: CYP450 unigenes identified in *R. glutinosa* transcriptomes; Supplementary Table S12: Putative dehydratase genes in *R. glutinosa* transcriptomes; Supplementary Table S13: Decarboxylase candidate genes in *R. glutinosa* transcriptomes; Supplementary Table S14: Putative hydroxylase and epoxidase genes involved in catalpol biosynthesis; Supplementary Table S15: Accession numbers of TPSs used for phylogenetic analysis in Figure 8.
