# Supplementary material for: Transcriptome Analysis Provides Insights into Catalpol Biosynthesis in the Medicinal Plant Rehmannia glutinosa and the Functional Characterization of RgGES Genes
Source: Genes (Basel). 2024 Jan 24;15(2):155. doi: 10.3390/genes15020155 (PMC10888080; doi:10.3390/genes15020155)
Supplement: Supplementary file 1 [file genes-15-00155-s001.zip › Supplementary Figures.pdf]

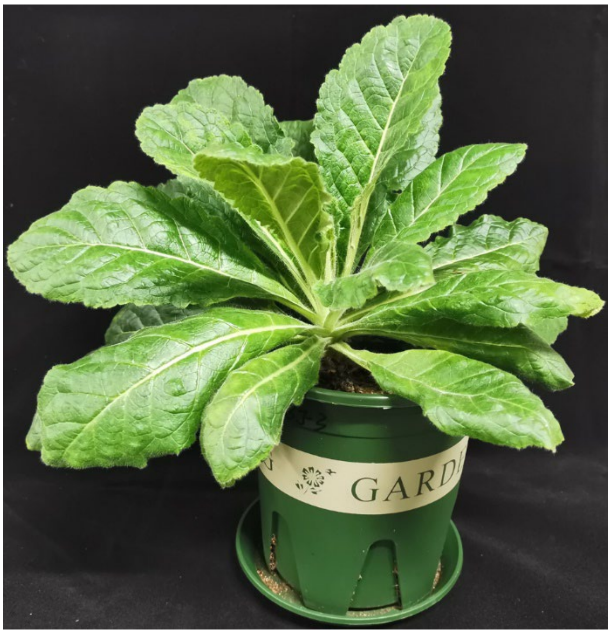

Supplementary Figure S1. The *R. glutinosa* plant.

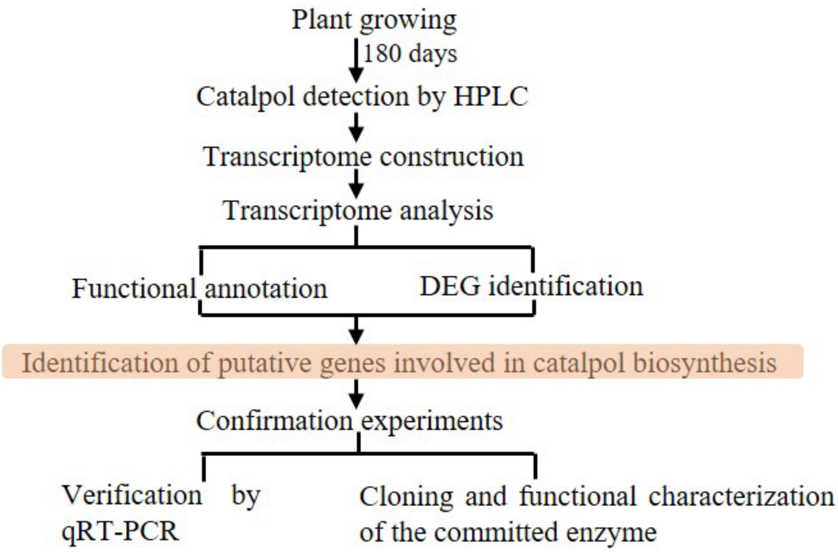

Supplementary Figure S2. The flowchart to the Material and Method section for summarizing methodology.

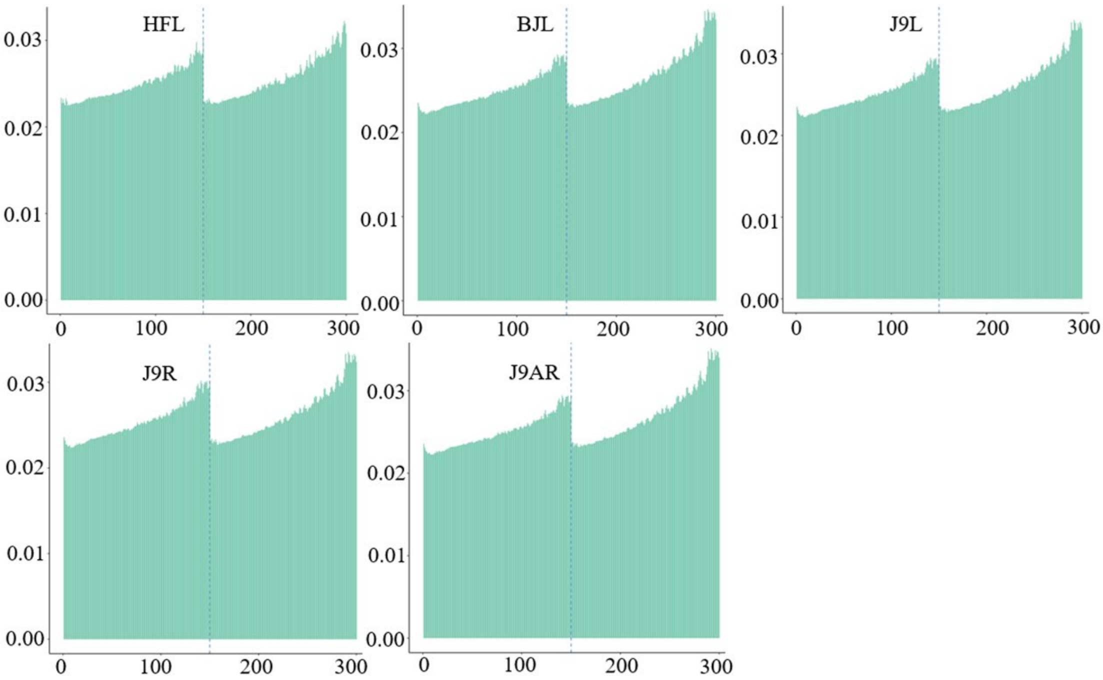

Supplementary Figure S3. The error rate distribution of unigenes.

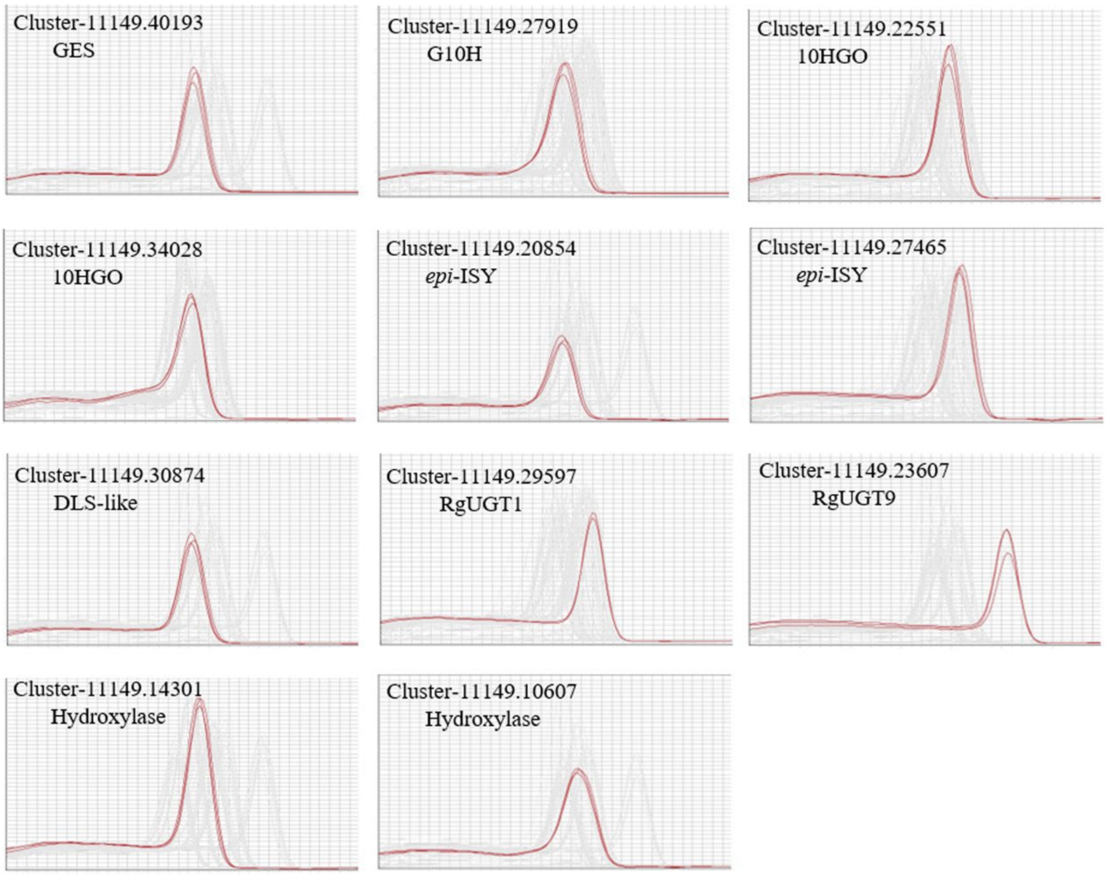

Supplementary Figure S4. The melting curves of the genes chosen for qRT-PCR analysis.

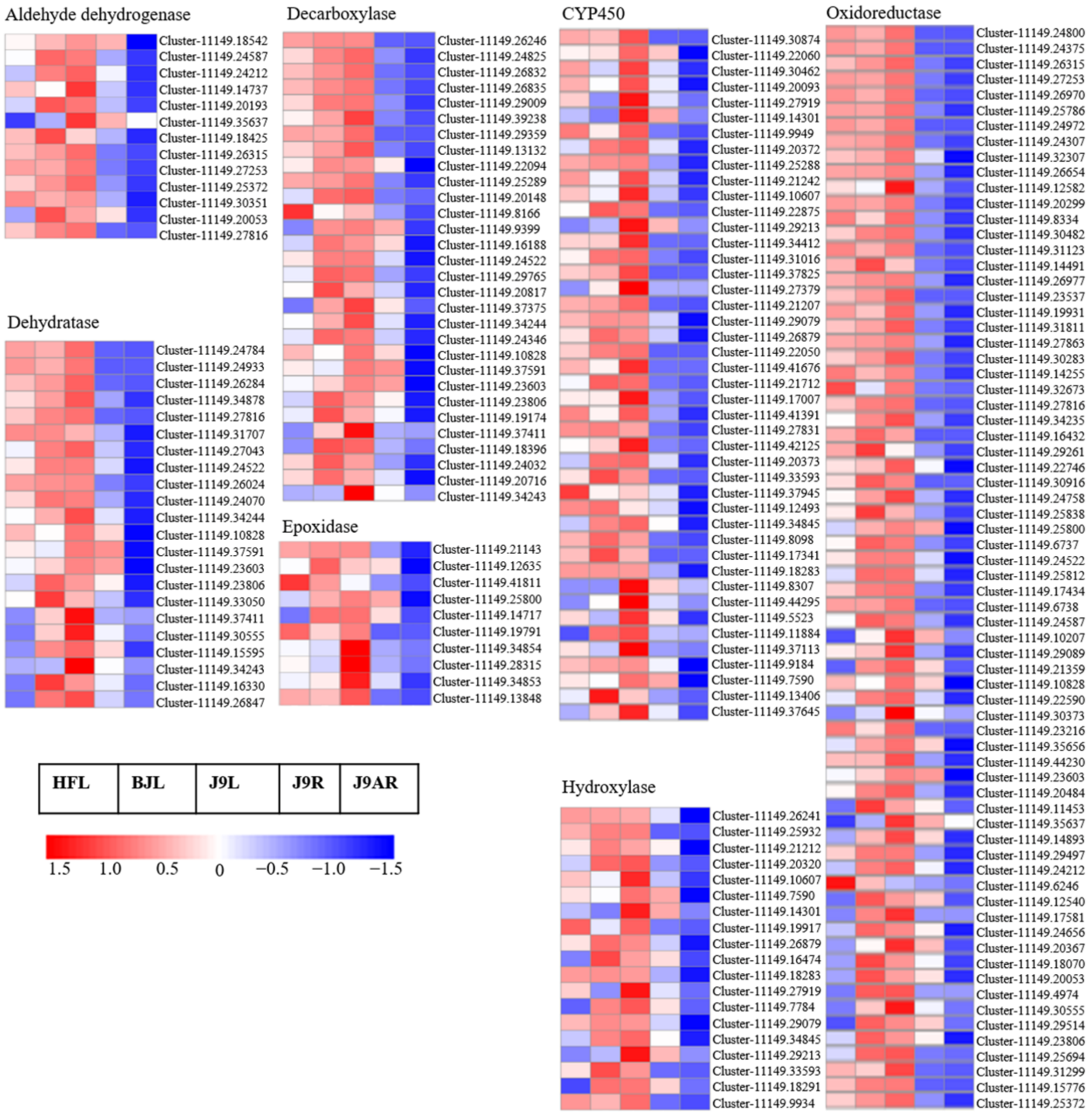

Supplementary Figure S5. The heatmap of differentially expressed genes that were evaluated in this study.

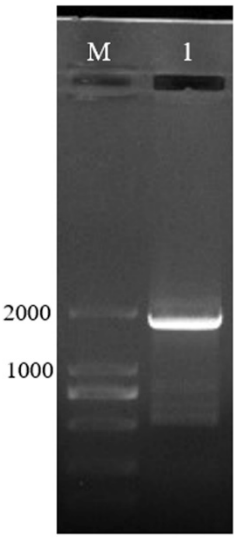

Supplementary Figure S6. Agarose gel electrophoresis for PCR products of *RgGES*. M, DNA marker; 1, PCR product of *RgGES*.

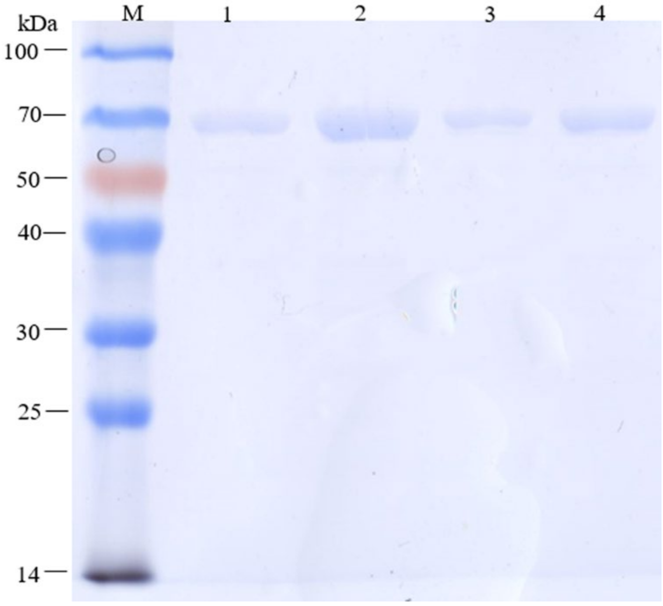

Supplementary Figure S7. The SDS-PAGE analysis of the recombinant RgGES1 and RgGES2 protein. M, protein marker; 1-2, the purified RgGES1 protein; 3-4, the purified RgGES2 protein.
