# Supplementary material for: Transcriptome Analysis Provides Insights into Catalpol Biosynthesis in the Medicinal Plant Rehmannia glutinosa and the Functional Characterization of RgGES Genes
Source: Genes (Basel). 2024 Jan 24;15(2):155. doi: 10.3390/genes15020155 (PMC10888080; doi:10.3390/genes15020155)
Supplement: Supplementary file 1 [file genes-15-00155-s001.zip › Supplementary Table S1.pdf]

Supplementary Table S1. Primers used in this study.

Gene Name	Gene number	Primer No.	Primer name	Sequence	Function
GES	Cluster-11149.40193	Primer 1	GES-TF	ATGGTGGAGGCGCAGTGGT	Real time PCR
		Primer 2	GES-TR	GCGGCTGAGAAGAGCTTAGGG	
G10H	Cluster-11149.27919	Primer 3	G10H-TF	TGTGGAATATCATGGTGGAG	Real time PCR
		Primer 4	G10H-TR	GTTTCTTTGGTGGCGGTGAG	
10HGO	Cluster-11149.34028	Primer 5	10HGO1-TF	ACGGTGAAATTCGCTAAGGC	Real time PCR
		Primer 6	10HGO1-TR	GTGGGATACCAACCAAGATAAG	
	Cluster-11149.22551	Primer 7	10HGO2-TF	CCATCTGGCTTTCTTTCC	Real time PCR
		Primer 8	10HGO2-TR	CCTTGTTGCCTACTTCTGTC	
	Cluster-11149.31508	Primer 9	10HGO3-TF	CAGTTCAGGGAAGACAAAC	Real time PCR
		Primer 10	10HGO3-TR	ATTCGGACCAAGTAGAGC	
ISY	Cluster-11149.20854	Primer 11	ISY1-TF	TCGAGGTGGAATATGAAGAG	Real time PCR
		Primer 12	ISY1-TR	ATCCGCAAACCAGAATGT	
	Cluster-11149.27465	Primer 13	ISY2-TF	CGAAATGGACAGCACGAACC	Real time PCR
		Primer 14	ISY2-TR	GCACGGCGAGAACCCGAATA	
DLS	Cluster-11149.30874	Primer 15	DLS-TF	CCGAACATTGCCGATTATTT	Real time PCR
		Primer 16	DLS-TR	TCCATCTTTCCCATCACCCT	
DLGT	Cluster-11149.29597	Primer 17	DLGT1-TF	GCCCGCCAGGTCCGTGATAT	Real time PCR
		Primer 18	DLGT1-TR	CCAACCGCTGTGCGTGAAA	
	Cluster-11149.23607	Primer 19	DLGT2-TF	AGTCCCGCTTCTCCGGTTAC	Real time PCR
		Primer 20	DLGT2-TR	CCTATCGCCGCCTTCACTAA	
DLH	Cluster-11149.14301	Primer 21	DLH1-TF	AGAAACGGCCTTGGGAGACT	Real time PCR
		Primer	DLH1-	CGTGACCATTGGCTGCTTTA	

		22	TR		
	Cluster-11149.10607	Primer 23	DLH2-TF	GAGCAGAGTATTTGCCATCGG	Real time PCR
		Primer 24	DLH2-TR	CCAAACCAGGAGATTAGAAGTG	
TIP	-	Primer 25	TIP-TF	ATGCGGCTGGAAGCAGGAAG	Real time PCR
		Primer 26	TIP-TR	GCCAACGCGACCATATCAA	
RgGES	Cluster-11149.40193	Primer 27	RgGES-FP	CGCGGATCCATGGCTTGTGCAACGAGCAACA	Cloning
		Primer 28	RgGES-RP	CGCGTCGACTCAAACAAGGGGAGTGAAGAAG	
