# Supplementary material for: Transcriptome Analysis Provides Insights into Catalpol Biosynthesis in the Medicinal Plant Rehmannia glutinosa and the Functional Characterization of RgGES Genes
Source: Genes (Basel). 2024 Jan 24;15(2):155. doi: 10.3390/genes15020155 (PMC10888080; doi:10.3390/genes15020155)
Supplement: Supplementary file 1 [file genes-15-00155-s001.zip › Supplementary Table S15.pdf]

Supplementary Table S15. Accession numbers of TPSs used for phylogenetic analysis in Figure 8.

Species	Terpene synthases	Accession numbers
Abies grandis	γ-humulene synthase	AAC05728.1
Arabidopsis thaliana	1,8-cineole synthase	AEE77075.1
Artemisia annua	8-*epi*-cedrol synthase	AAF80333.1
Artemisia annua	β-caryophyllene synthase	AAL79181.1
Abies grandis	α-bisabolene synthase	AAC24192.1
Oryza sativa	linalool synthase	ACF05530.1
Helianthus annuus	germacrene A synthase 2	ABY49939.1
Helianthus annuus	cadinene synthase	ACA33926.1
Mentha spicata	limonene synthase	AGN90912.1
Nicotiana tabacum	5-*epi*-aristolochene synthase	AAA19216.1
Oryza sativa	ent-copalyl diphosphate synthase	AAT11021.1
Picea sitchensis	ent-copalyl diphosphate synthase	ADB55709.1
Populus fremontii	isoprene synthase	AEK70967.1
Picea sitchensis	pinene synthase	AAP72020.1
Solidago canadensis	(+)-germacrene D synthase	AAR31144.1
Vitis vinifera	(*E*)-β-ocimene/myrcene synthase	ADR74206.1
Vitis vinifera	(+)-α-phellandrene synthase	ADR74201.1
Arabidopsis thaliana	terpenoid cyclase	AEE36246.1
Arabidopsis thaliana	(*S*)- (+)-linalool synthase	Q84UV0.2
Ocimum basilicum	geraniol synthase	AAR11765.1
Catharanthus roseus	geraniol synthase	AFD64744.1
