# Supplementary material for: Transcriptome Analysis Provides Insights into Catalpol Biosynthesis in the Medicinal Plant Rehmannia glutinosa and the Functional Characterization of RgGES Genes
Source: Genes (Basel). 2024 Jan 24;15(2):155. doi: 10.3390/genes15020155 (PMC10888080; doi:10.3390/genes15020155)
Supplement: Supplementary file 1 [file genes-15-00155-s001.zip › Supplementary Table S2.pdf]

Supplementary Table S2. Transcriptome assembly statistics for *R. glutinosa*.

Item	Number
Number of unigenes	71142
Average length	1131
Median length	707
N50	1732
Total size of unigenes	80429218
Longest unigene length	15639
Shortest unigene length	301
