# Supplementary material for: Transcriptome Analysis Provides Insights into Catalpol Biosynthesis in the Medicinal Plant Rehmannia glutinosa and the Functional Characterization of RgGES Genes
Source: Genes (Basel). 2024 Jan 24;15(2):155. doi: 10.3390/genes15020155 (PMC10888080; doi:10.3390/genes15020155)
Supplement: Supplementary file 1 [file genes-15-00155-s001.zip › Supplementary Table S8.pdf]

Supplementary Table S8. Accession numbers of PSPGs used for phylogenetic analysis in Figure 6.

Name	Species	Functions	Accession No.
Cm1,2RhaT	*Citrus maxima*	Flavanone 7-O-glucoside 1,2-O-rhamnosyltransferase	AAL06646
UGT94D1	*Sesamum indicum*	Sesaminol 2-O-glucoside	BAF99027
BpUGAT	*Bellis perennis*	Anthocyanidin 3-O-glucoside 1,2-O-glucosyltransferase	BAD77944
In3GGT	*Ipomoea nil*	Anthocyanidin 3-O-glucoside 2”-O-glucosyltransferase	AB192314
6RhaT	*Petunia hybrida*	Anthocyanidin 3-O-glucoside 1,6-O-rhamnosyltransferase	CAA50376
ZOG1	*Phaseolus lunatus*	Zeatin O-glucosyltransferase	AF101972
ZOX1	*Phaseolus lunatus*	Zeatin O-xylosyltransferase	AF116858
UGT72E2	*Arabidopsis thaliana*	Oniferylalcohol glucosyltransferase	AB018119
UGT72B1	*Arabidopsis thaliana*	Hydroxybenzoic acid glucosyltransferase	CAB80916
UGT71G1	*Medicago truncatula*	Flavonoid 3’-O-glucosyltransferase	AAW56092
UGT71C1	*Arabidopsis thaliana*	Hydroxycinamate, flavonoid 3-O-glucosyltransferase	AC005496
NtGT1a	*Nicotina tabacum*	Glucosyltransferase with broad substrate specificity	AB052558
NtGT3	*Nicotina tabacum*	Glucosyltransferase with broad substarate specificity	AB072918
UGT73C5	*Arabidopsis thaliana*	Zeatin O-glucosyltransferase	AAD20156
SaGT4a	*Solanum aculeatissimum*	Seroidal sapogenin 3-O-glucosyltransfera	BAD89042
B5GT	*Dorotheanthus*	Betanidin 5-O-glucosyltransferase	Y18871
CaUGT2	*Catharanthus roseus*	Curcumin glucosyltransferase	AB159213
F7GT	*Scutellaria*	Flavonoid 7-O-	BAA83484

	baicalensis	glucosyltransferase	
UGT75B1	*Arabidopsis thaliana*	Indole 3-acetic acid, sinapic acid glucosyltransferase	AAF79370
NtGT2	*Nicotina tabacum*	Flavonol 7-O-coumarin 3- O-glucosyltransferase	AB072919
UGTCs2	*Crocus sativus*	Crocetin glucosyltransgerase	AAP94878
NtSAGT	*Nicotina tabacum*	Salicylic acid glucosyltransferase	AAF61647
UGT74B1	*Arabidopsis thaliana*	Thiohydroximate S-glucosyltransferase	AC002396
UGT76G1	*Stevia rebaudiana*	Stevioside glucosyltransferase	AAR06912
UGT76C1	*Arabidopsis thaliana*	Trans-zeatin N-glucosyltransferase	BAB10792
CrUGT7	*Catharanthus roseus*	Glucosyltransferase with broad substrate specificity	AB733666
ZeaBx8	*Zea mays*	Benzoxazinoid 2-O-glucosyltransferas	AAL57037
UGT85B1	*Sorghum bicolor*	p-Hydroxymandelonitrile O-glucosyltransferase	AAF17077
CrUGT8	*Catharanthus roseus*	Iridoid 1-O-glucosyltransferase	AB733667
LjUGT12	*Lonicera japonica*	Iridoid 1-O-glucosyltransferase	
UGT85A1	*Arabidopsis thaliana*	Trans-zeatin O-glucosyltransferase	AAF18537
CrUGT6	*Catharanthus roseus*	Iridoid 1-O-glucosyltransferase	AB591741
GjUGT2	*Gardenia jasminoides*	Iridoid 1-O-glucosyltransferase	AB555732
UGT78D1	*Arabidopsis thaliana*	Flavonol 3-O-glicoside L-rhamnosyltransferase	AC009917
VvGT1	*Vitis vinifera*	Flavonoid 3-O-glucosyltransferase	AAB81682
UGT85A84	*Osmanthus fragrans*	glucosyltransferase	AYN74351.1
UGT85A3	*Arabidopsis thaliana*	UDP-glucosyl transferase	AEE30236.1
